# Free Association Database for a 62-Word Dataset Including Emotion and Colour Terms in English, Estonian, French, German, Italian, Lithuanian, and Spanish: Data from 14 Countries

**DOI:** 10.5334/jopd.140

**Published:** 2025-08-04

**Authors:** Domicele Jonauskaite, Déborah Epicoco, Maliha Bouayed Meziane, Britt Burton, Violeta Corona, Eduardo Fonseca-Pedrero, Consuelo González-Dávila, Jelena Havelka, Eric Laurent, Bigna Lenggenhager, Tobias Loetscher, Stephanie Lopez Castiñeira, Leila Manni, Philip Mefoh, Daniel Oberfeld, Merle Oguz, Rabia Yağmur Özduran, Corinna Perchtold-Stefan, Patricia Quant, Michael Quiblier, Maja Roch, Sude Sarayköylü, Giulia F. M. Spagnulo, Maël Theubet, Cecilia Toscanelli, Mari Uusküla, Christine Mohr

**Affiliations:** 1 Institute of Psychology, University of Lausanne, Lausanne, Switzerland; 2 Faculty of Psychology, University of Vienna, Vienna, Austria; 3 School of Social Work, University of Applied Sciences and Arts of Western Switzerland (HETSL | HES-SO), Lausanne, Switzerland; 4 Preparatory Classes Department, École Nationale Polytechnique, Algiers, Algeria; 5 Justice and Society, University of South Australia, Adelaide, Australia; 6 Facultad de Ciencias Económicas y Empresariales, Universidad Panamericana, Zapopan, Mexico; 7 Department of Educational Sciences, University of La Rioja, Logroño, Spain; 8 Faculty of Psychology, National Autonomous University of Mexico (UNAM), Mexico City, Mexico; 9 School of Psychology, University of Leeds, Leeds, UK; 10 UMR INSERM 1322 LINC & UAR 3124 CNRS MSHE Ledoux, Université Marie et Louis Pasteur, Besançon, France; 11 Department of Psychology, University of Zurich, Zurich, Switzerland; 12 Association for Independent Research, Zurich, Switzerland; 13 Department of Psychology, University of Konstanz, Konstanz, Germany; 14 Department of Psychology, University of Nigeria, Nsukka, Enugu State, Nigeria; 15 Institute of Psychology, Johannes Gutenberg University Mainz, Mainz, Germany; 16 School of Humanities, Tallinn University, Tallinn, Estonia; 17 Faculty of Human and Social Sciences, Yasar University, Izmir, Turkey; 18 Department of Psychology, University of Graz, Graz, Austria; 19 Faculty of Life Sciences, University of Vienna, Vienna, Austria; 20 Department of Developmental Psychology and Socialization, University of Padova, Padova, Italy; 21 Department of Psychology, Carl von Ossietzky University Oldenburg, Oldenburg, Germany; 22 Institute of Psychology, University of Bern, Bern, Switzerland; 23 Research Group Work & Organisational Psychology (WOPP-O2L), KU Leuven, Leuven, Belgium

**Keywords:** Semantic networks, Cross-linguistic semantics, Cross-cultural psychology, Multilingual database, Psycholinguistics

## Abstract

This article presents a free association database containing responses to 62 stimulus words (including colour terms, emotion words, and common nouns) across seven languages: English, Estonian, French, German, Italian, Lithuanian, and Spanish. Data were collected online from 1,439 participants (mean age 31.47 years) across 14 countries, yielding 223,786 responses. All data were cleaned, normalised, and organised for analysis, with both raw and processed datasets available on OSF. This cross-linguistic resource enables research on semantic networks, psycholinguistics, translation studies, and cross-cultural comparisons, providing insights into how meaning is constructed within and across different languages and cultures.

## 1. Background

The open data movement has gained momentum in psychology over the past decade, responding to the field’s ongoing concerns about reproducibility (Munafò et al., 2017; L. D. Nelson et al., 2018). In this regard, research depends on robust, accessible datasets that enable replication, facilitate study design, and allow for novel methodologies and analyses to be run in new populations. Making psychological datasets truly Findable, Accessible, Interoperable, and Reusable (FAIR; Wilkinson et al., 2016) is crucial for advancing research and addressing reproducibility concerns (Munafò et al., 2017; L. D. Nelson et al., 2018). Despite the fundamental scientific value of shared data (Meyer, 2018), many datasets remain either completely unpublished or isolated in lab-specific repositories, where the lack of documentation and standardisation limit their utility. This data article contributes a new, cross-linguistic resource designed with FAIR principles at core.

Over recent years, there have already been notable endeavours to provide researchers with extensive datasets, focused on language and cognition (e.g., Bradley & Lang, 1999; Brysbaert et al., 2014; Brysbaert & New, 2009; De Deyne et al., 2019; Duyck et al., 2004; Keuleers et al., 2012; Kuperman et al., 2012; Lenci et al., 2013; Lynott et al., 2020; Monnier & Syssau, 2017; D. L. Nelson et al., 2004; Stadler et al., 1999; Sutton & Altarriba, 2016; Warriner et al., 2013). These datasets are important because well-designed studies require well-controlled linguistic material, which is effortful to collect. Word sets are commonly controlled for word frequency, length, imageability, valence, arousal, age of acquisition, and knowledge of other languages (e.g., Brysbaert et al., 2016, 2018; Keuleers et al., 2015; Kuperman et al., 2014; Warriner & Kuperman, 2015). When a subsequent study is conducted in another language, these norms quickly change, requiring new norms and likely also new word sets (e.g., Willemin et al., 2016).

Challenges are also high for semantic association studies, in which the structure of knowledge and abstract representation are commonly tested by having people spontaneously report what comes to their mind when being presented with a trigger stimulus. Already Sigmund Freud (1913) used free associations to understand his patients’ minds by having them freely express emerging thoughts (also see, Lothane, 2018). In clinical settings, free associations remain in regular use (for an overview, see Novac & Blinder, 2021). They are also popular in experimental fields, such as creativity (Merseal et al., 2025), language learning (Citraro et al., 2023; Mak & Twitchell, 2020), cognition (De Deyne et al., 2013; Muraki & Pexman, 2024), and computational modelling of semantic networks (Steyvers & Tenenbaum, 2005). Indeed, word associations are thought to reflect the underlying structure of human semantic memory and provide a window into how meaning is constructed within and across languages and cultures (D. L. Nelson et al., 2000).

Large-scale word association datasets have been published for some languages, such as English (De Deyne et al., 2019), Dutch (De Deyne & Storms, 2008), or Spanish (Cabana et al., 2023). However, we know of no comprehensive dataset on semantic associations for the same word set in different languages. Due to our interest in the cross-cultural links between colour and emotion (e.g., Jonauskaite et al., 2019, 2020, 2024; Jonauskaite & Mohr, 2025; Ram et al., 2020; Uusküla et al., 2023; Weijs et al., 2023), we had collected free association data for colour terms, emotion terms, and various ‘filler’ words in English, Estonian, French, German, Italian, Lithuanian, and Spanish.

Here, we present this complete dataset. It contains 223,786 free association responses from 1,439 participants across 14 countries who provided associations to 62 stimulus words in seven languages. The stimulus words comprise 11 basic colour terms (e.g., *red, green, blue*), 5 non-basic colour terms (e.g., *turquoise, beige, violet*), 20 emotion terms (e.g., *love, joy, sadness, anger*), and 26 common nouns, the latter including eight animals (e.g., *cat, dog, giraffe, elephant*), six domestic objects (e.g., *basket, nail, corridor*), six environmental elements (e.g., *cloud, liquid, poison*), and six abstract concepts (e.g., *mathematics, peace, symbol*). We publish this association dataset alongside demographic variables including age, gender, profession, country of origin, country of residence, and language fluency.

## 2. Material and Method

### 2.1. Study Design

We collected free associations in an online questionnaire, using the LimeSurvey platform (LimeSurvey Project Team, 2020). Each participant saw all 62 stimulus words (see Stimuli) in one of the seven languages (English, Estonian, French, German, Italian, Lithuanian, and Spanish). Participants were instructed to note the first three words that came to their mind when they thought of this stimulus word. Most participants produced individual words, but some also produced short phrases. Some participants produced fewer than three responses and others more. Overall, the dataset contains within-subject qualitative data (i.e., word responses), which we cleaned and normalised (see Data Preparation). Thus, they are ready for further use.

### 2.2. Time of Data Collection

The data were collected in the following time intervals: i) 30^th^ October 2019 – 8^th^ July 2020; ii) 12^th^ November 2020 – 20^th^ October 2021, and iii) 17^th^ January 2023 – 2^nd^ March 2023.

### 2.3. Locations of Data Collection

The formal data collection was coordinated at the University of Lausanne, Switzerland by DE, DJ, and CM. Participant recruitment from different countries and places were realised by the co-authors in 14 different countries:

– Algeria, Algiers (MB)
– Australia, Adelaide (BB and TL)
– Austria, Graz (CPS) and Vienna (DJ and RYÖ)
– Estonia, Tallinn (MU)
– France, Besançon (EL)
– Germany, Mainz (DO)
– Italy, Padua (MR and CT)
– Lithuania, Kaunas (DJ)
– Mexico, Guadalajara (VC)
– Nigeria, Nsukka (PM)
– Spain, Logroño (EFP)
– Switzerland, Lausanne (DE, DJ, and CM) and Zurich (BL)
– UK, Leeds (JH)
– USA, Boise (MQ)

### 2.4. Sampling, Sample, and Data Collection

We recruited 1,439 participants – 1,141 women, 290 men, eight participants did not report their gender. We recruited participants of a wide age range resulting in a mean age of 31.47 years (*SD* = 13.76 years, range = 16–80 years). With data extraction, we ensured that participants were native or highly fluent speakers of the language they had completed the survey in. We obtained data in seven languages: English, Estonian, French, German, Italian, Lithuanian, and Spanish (see Table 1 for demographic data per language).

**Table 1:** Demographic information of the studied participants.


SURVEY LANGUAGE	COUNTRY OF ORIGIN	COUNTRY OF RESIDENCE	*N*				AGE (YEARS)		
	
			TOTAL	MEN	WOMEN	OTHER	*MEAN*	*SD*	RANGE

English	Australia, UK, USA, Nigeria, and other countries	Australia, UK, USA, Nigeria, and other countries	241	59	180	2	37.78	15.17	18–80

Estonian	Estonia	Estonia and other countries	123	10	113	0	43.75	14.15	20–74

French	Switzerland, France, Algeria, Portugal, and other countries	Switzerland, France, Algeria, and other countries	316	49	266	1	23.87	9.50	18–74

German	Germany, Austria, Switzerland, and other countries	Austria, Germany, Switzerland, and other countries	357	69	285	3	25.59	8.64	18–62

Italian	Italy and other countries	Italy and other countries	56	14	42	0	32.35	11.40	19–57

Lithuanian	Lithuania and other countries	Lithuania and other countries	179	29	150	0	41.73	13.70	16–69

Spanish	Mexico, Spain, and other countries	Mexico, Spain, and other countries	167	60	105	2	31.97	12.81	18–71


*Note*. Countries of origin and of residence are ordered by decreasing sample size (from biggest to smallest). In both cases, we explicitly named countries that had at least 20 participants. ‘Other countries’ constituted fewer than 20 participants per country in the respective survey language.

Participants came from different countries, with the most common countries being Switzerland (*n* = 235), Lithuania (*n* = 178), Germany (*n* = 152), Estonia (*n* = 122), Austria (*n* = 109), Mexico (*n* = 87), Spain (*n* = 79), Australia (*n* = 63), Italy (*n* = 58), USA (*n* = 58), Algeria (*n* = 57), UK (*n* = 51), France (*n* = 50), and Nigeria (*n* = 47). The remaining 93 participants came from other countries or indicated two countries (e.g., France and Switzerland) as countries of origin. Most younger participants were university students, while the remaining participants had diverse occupations, all of which appear in the dataset. Participation was voluntary. Participants did not receive monetary remuneration. Some student participants in Switzerland, Germany, and Austria received course credits for taking part in the study.

### 2.5. Material

#### 2.5.1. Stimuli

We selected 62 words as stimuli, falling into the four following domains: i) 11 basic colour terms (Table 2), ii) 5 non-basic colour terms (Tables 2 and 3), iii) 20 emotion terms (Table 2), and iv) 26 ‘filler’ words (i.e., animal names, domestic items, environmental elements, abstract concepts; Table 2).

**Table 2:** Stimulus words in seven languages – direct translations across the studied languages.


WORD TYPE	ENGLISH	ESTONIAN	FRENCH	GERMAN	ITALIAN	LITHUANIAN	SPANISH

Basic colour term	Red	Punane	Rouge	Rot	Rosso	Raudona	Rojo

Basic colour term	Orange	Oranž	Orange	Orange	Arancione	Oranžinė	Naranja

Basic colour term	Yellow	Kollane	Jaune	Gelb	Giallo	Geltona	Amarillo

Basic colour term	Green	Roheline	Vert	Grün	Verde	Žalia	Verde

Basic colour term	Blue	Sinine	Bleu	Blau	Blu	Mėlyna	Azul

Basic colour term	Purple	Lilla	Violet	Lila	Viola	Violetinė	Violeta

Basic colour term	Pink	Roosa	Rose	Rosa	Rosa	Rožinė	Rosado

Basic colour term	Brown	Pruun	Brun	Braun	Marrone	Ruda	Marrón

Basic colour term	White	Valge	Blanc	Weiß	Bianco	Balta	Blanco

Basic colour term	Grey	Hall	Gris	Grau	Grigio	Pilka	Gris

Basic colour term	Black	Must	Noir	Schwarz	Nero	Juoda	Negro

Non-basic colour term	Turquoise	Türkiissinine	Turquoise	Türkis	Turchese	Turkio spalva	Turquesa

Non-basic colour term	Beige	Beež	Beige	Beige	Beige	Smėlinė	Beige

Emotion term	Interest	Huvi	Intérêt	Interesse	Interesse	Susidomėjimas	Interesar

Emotion term	Amusement	Lõbu	Amusement	Belustigung	Divertimento	Linksmumas	Diversión

Emotion term	Pride	Uhkus	Fierté	Stolz	Fierezza	Išdidumas	Orgullo

Emotion term	Joy	Rõõm	Joie	Freude	Gioia	Džiaugsmas	Alegría

Emotion term	Contentment	Rahulolu	Contentement	Zufriedenheit	Soddisfazione	Pasitenkinimas	Contento

Emotion term	Admiration	Imetlus	Admiration	Bewunderung	Ammirazione	Žavėjimasis	Admiración

Emotion term	Love	Armastus	Amour	Liebe	Amore	Meilė	Amor

Emotion term	Relief	Kergendus	Soulagement	Erleichterung	Sollievo	Nusiraminimas	Alivio

Emotion term	Compassion	Kaastunne	Compassion	Mitgefühl	Compassione	Užuojauta	Compasión

Emotion term	Pleasure	Nauding	Plaisir	Vergnügung	Piacere	Malonumas	Placer

Emotion term	Sadness	Kurbus	Tristesse	Trauer	Tristezza	Liūdesys	Tristeza

Emotion term	Guilt	Süü	Culpabilité	Schuld	Colpevolezza	Kaltė	Culpa

Emotion term	Regret	Kahetsus	Regret	Bereuen	Rimpianto	Apgailestavimas	Arrepetimiento

Emotion term	Shame	Häbi	Honte	Scham	Vergogna	Gėda	Vergüenza

Emotion term	Disappointment	Pettumus	Déception	Enttäuschung	Delusione	Nusivylimas	Decepción

Emotion term	Fear	Hirm	Peur	Angst	Paura	Baimė	Miedo

Emotion term	Disgust	Vastikus	Dégoût	Ekel	Disgusto	Pasibjaurėjimas	Asco

Emotion term	Contempt	Põlgus	Mépris	Verachtung	Disprezzo	Panieka	Desprecio

Emotion term	Hate	Vihkamine	Haine	Hass	Odio	Neapykanta	Odio

Emotion term	Anger	Viha	Colère	Wut	Rabbia	Pyktis	Ira

Other	Basket	Korv	Panier	Korb	Cesto	Krepšys	Cesta

Other	Cheese	Juust	Fromage	Käse	Formaggio	Sūris	Queso

Other	Cloud	Pilv	Nuage	Wolke	Nuvola	Debesis	Nube

Other	Liquid	Vedelik	Liquide	Flüssigkeit	Liquido	Skystis	Líquido

Other	Nail	Küüs	Ongle	Nagel	Unghia	Nagas	Uña

Other	Cat	Kass	Chat	Katze	Gatto	Katė	Gato

Other	Dog	Koer	Chien	Hund	Cane	Šuo	Perro

Other	Horse	Hobune	Cheval	Pferd	Cavallo	Arklys	Caballo

Other	Domestic	Kodune	Domestique	Häuslich	Domestico	Naminis	Doméstico

Other	Hood	Kapuuts	Capuche	Kaputze	Cappuccio	Kapišonas	Capucha

Other	Routine	Rutiin	Routine	Routine	Routine	Rutina	Rutina

Other	Symbol	Sümbol	Symbole	Symbol	Simbolo	Simbolis	Símbolo

Other	Corridor	Koridor	Couloir	Korridor	Corridoio	Koridorius	Pasillo

Other	Peace	Rahu	Paix	Frieden	Pace	Taika	Paz

Other	Ladder	Redel	Echelle	Leiter	Scala	Kopėčios	Escalera

Other	Elephant	Elevant	Eléphant	Elefant	Elefante	Dramblys	Elefante

Other	Dizzy	Uimane	Etourdi	Schwindelig	Stordito	Apsvaigęs	Mareado

Other	Poison	Mürk	Poison	Gift	Veleno	Nuodai	Veneno

Other	Hay	Hein	Foin	Heu	Fieno	Šienas	Heno

Other	Mathematics	Matemaatika	Mathématiques	Mathematik	Matematica	Matematika	Matemáticas

Other	Giraffe	Kaelkirjak	Girafe	Giraffe	Giraffa	Žirafa	Jirafa

Other	Squirrel	Orav	Écureuil	Eichhörnchen	Scoiattolo	Voverė	Ardilla

Other	Echo	Kaja	Écho	Echo	Eco	Aidas	Eco

Other	Bean	Uba	Haricot	Bohne	Fagiolo	Pupelė	Alubia

Other	Mouse	Hiir	Souris	Maus	Topo	Pelė	Ratón

Other	Tiger	Tiiger	Tigre	Tiger	Tigre	Tigras	Tigre


**Table 3:** Remaining stimulus words (mostly non-basic colour terms), in seven languages – not direct translations across the studied languages. See explanations in text for the inclusion of *Geld* as a stimulus word in German.


ENGLISH	ESTONIAN	FRENCH	GERMAN	ITALIAN	LITHUANIAN	SPANISH

Lilac	Violetne	Lilas	Violett	Lilla	Alyvinė	Lila

Violet	Purpur	Pourpre	Purpur	Porpora	Purpurinė	Púrpura

Maroon	Vesihall	Marron	Ocker	Azzuro	Žydra	Rosa

			Geld			


We took the basic colour terms from colour naming studies (Del Viva et al., 2023; Forbes, 2006; Lindsey & Brown, 2014; Uusküla & Bimler, 2016; Vejdemo et al., 2015) and the 20 emotion terms from the Geneva Emotion Wheel (Scherer, 2005; Scherer et al., 2013). Translations for the basic colour and emotion terms were adopted from Jonauskaite et al. (2020). The five non-basic colour terms were chosen individually for each language based on previous studies (see Lindsey & Brown, 2014; Morgan, 1993; Mylonas & MacDonald, 2016; Uusküla & Bimler, 2020). Two words (i.e., *turquoise* and *beige*) were translated across the seven languages while three non-basic words, listed in Table 3 (four in German), could vary across the languages. The ‘filler’ words were taken from Fitzpatrick and colleagues (2015), which two bilingual speakers translated following the back-translation procedure.

During the data collection, we realised that the German word for *yellow* was displayed incorrectly in our survey – *Geld* (meaning ‘money’) instead of *Gelb* (meaning ‘yellow’). We corrected the error and collected an additional sample of German speakers. This is why we have German data on 63 stimulus words (both *Geld* and *Gelb*), but the number of participants responding to *Geld* and *Gelb* is smaller than to the remaining words. Put differently, all participants responded to the same 61 words. Additionally, some participants responded to *Geld* and others to *Gelb*.

#### 2.5.2. Procedure

Data collection was conducted by co-authors, who widely distributed the link to the online survey that opened directly in their target language (i.e., the national language of their country). The PDF versions of the surveys in all seven languages are accessible in our repository (see Object and File Names).

The survey started by stating its main goal and providing ethical information (i.e., participation was anonymous and confidential; responses were to be used for research purposes only; participants could stop the survey at any time). Participants provided their consent by clicking on the ‘Next’ button. After collecting some demographic information (age, gender, profession, country of origin, country of residence, native language and fluency in the language of the survey), participants read the following instructions:

On the screen, you will see one word after the other. For each word, please write down the first three words that come to your mind. For example, you see the word SUN. Then, SKY, YELLOW, and BEAUTIFUL might be the first words that come to your mind. In that case, you would write these words into the word field. There are no right or wrong answers, we are interested in your personal opinion.

Then, participants clicked on the ‘yes’ button to confirm they understood the task and were ready to continue. In the main part of the survey, they sequentially saw the 62 stimulus words, presented in a semi-randomised order, meaning the three words for *purple, violet* and *lilac* never followed each other (due to an a priori research interest, see Epicoco et al., 2021, 2024). For each stimulus word, participants were asked to write down three responses that spontaneously came to their minds when thinking about that word (i.e., three associations). Requesting three associations per stimulus word was intended to elicit a more diverse and heterogeneous set of responses (also see, De Deyne et al., 2013). Participants wrote down the responses in a single response box, with some providing fewer and others providing more than three responses. Everyone provided at least one response per stimulus word. Missing responses were coded as ‘NA’ in the datasheets. Participants were then thanked and debriefed at the end. They were also given contact details in case they wished to get in touch. The study took around 22 minutes to complete.

### 2.6. Quality Control

We implemented several quality control measures throughout the data collection and processing phases to ensure the reliability and validity of our dataset. These measures aimed to produce a robust and reliable free association dataset for cross-linguistic research, with careful attention paid to maintaining both language-specific authenticity and cross-linguistic comparability.

#### 2.6.1. Participant Screening and Selection

We asked participants to complete the survey in the language they were highly proficient in. We verified language proficiency by asking all participants to self-report their language fluency on an 8-point scale (1 = not at all, 8 = completely fluent). We excluded participants who reported fluency scores of 6 or lower in the language they completed the survey in. Thus, we arrived at the mean language fluency score of 7.97 (*SD* = 0.18).^1^ We also excluded participants, whose responses did not match the language of the survey (e.g., most responses given in English, when the language of the survey is Lithuanian). If participants produced just a couple of responses in a different language (e.g., *touché* in English), we kept those participants. Finally, during the cleaning process, we ensured that all participants with non-sensical responses were excluded too (e.g., typing *xxx, sss, ddd*, etc.). Together, we had excluded 98 participants, whose data can be found in the raw data file (see Object and File Names).

#### 2.6.2. Survey Design and Implementation

The survey design incorporated several features to ensure consistent data collection across languages. We used a standardised LimeSurvey template (LimeSurvey Project Team, 2020) that was identical across all seven languages, with only the language of presentation differing. Instructions were carefully translated by native speakers and verified by the project coordinators to ensure conceptual equivalence across the surveys. For the presentation of stimulus words, we maintained a semi-randomised order across all languages to control for order effects, particularly ensuring that semantically related colour terms (*purple, violet, lilac*) never appeared consecutively. The survey platform required participants to provide at least one association for each stimulus word before proceeding, thereby ensuring no stimulus word was left entirely without a response.

#### 2.6.3. Data Cleaning and Normalisation

Extracting the raw data from the LimeSurvey, we noticed that participant responses for each stimulus word were placed in a single cell. Since most participants provided three responses per stimulus word, we had to separate them. Unfortunately, participants used different ways to indicate the separation between the responses: commas, semicolons, dashes, or simply spaces. Thus, we wrote a custom R code to automatically separate participant responses into different lines (see Object and File Names). Now, each word appeared in a new line, but it meant that multiple word responses (e.g., *summer holidays*) or longer phrases (e.g., *La vie en rose*) were separated too.

Therefore, in the subsequent manual validation step, highly fluent speakers of each language were tasked to put the separated words back into phrases. Afterwards, they cleaned and normalised the responses in the following way:

– **Typo correction**. We corrected words that were spelt incorrectly, unless we could not determine the intended word. In those cases, we left the responses as produced by the participants.
– **Preference for lowercase letters**. We ensured that all the responses were written in lowercase letters unless an uppercase letter was required by the orthography rules of that language (e.g., proper names in English, all nouns in German, etc.).
– **Normalisation of spelling**. In English, we converted American English responses to British English (*gray* became *grey*). We also corrected regional spelling to the national spelling. The latter was most pertinent in German, and we used Duden (https://www.duden.de) as the authority. Words that had multiple accepted spellings (e.g., *meow, miaow*, and *meaw* in English) were written in the most common word form.
– **Preference for singular word forms**. When appropriate, we normalised the noun and adjective responses to appear in a singular form (e.g., *cherries* became *cherry*). However, in some cases it was not appropriate because the plural word form was more frequent or the only one correct. In those cases, we kept all responses in plural (e.g., *eyes, trousers, shoes*).
– **Preference for nouns with no articles**. When appropriate, we deleted articles for nouns. Thus, in English, *the apple* became *apple*, in French, *l’histoire* became *histoire*. We did not delete possessive pronouns (e.g., *my dog*).
– **Preference for nouns in nominative grammatical case**. When appropriate, we normalised noun responses by choosing the nominative case. For instance, in Lithuanian, *atostogoms* became *atostogos*; in German, *des Kindes* became *Kind*; in English, *for peace* became *peace*.
– **Normalisation of adjectives**. Adjectives that occurred without nouns were normalised to appear in the ground form, which sometimes coincided with the masculine form. For instance, in French, *petite* became *petit*, in German, *schnelles* became *schnell*, in Lithuanian, *graži* became *gražu*, etc. Adjectives that occurred with nouns remained accorded to them (e.g., *salle vide, weiße Fahne, maža mergaitė*).
– **Normalisation of verbs**. Verbs that occurred alone were normalised to appear in the infinitive form. For instance, in German *schlafe* became *schlafen*, in Lithuanian, *važiuoju* became *važiuoti*.

Eleven fluent speakers were involved in total in this task, with 1–4 speakers per language. The entire process was supervised by the project coordinators (DE and DJ), ensuring homogeneity across the languages. When uncertain, speakers discussed the specific instances with each other and/or the project coordinators, favouring standard dictionary spelling and grammar rules.

#### 2.6.4. Documentation and Transparency

To facilitate quality assessment by future users, we share all versions of the data (raw, partially processed, and fully cleaned). We created comprehensive codebooks with clear explanations of all variables and processing steps (see FAIR Data and Codebook). We documented all exclusion criteria and their application to ensure reproducibility.

### 2.7. Data Anonymisation and Ethical Issues

The study was conducted in accordance with the ethical guidelines (World Medical Association, 2013) and received approval from the ethics committee at the Faculty of Social and Political Sciences, University of Lausanne (C_SSP_032020_00003). All data were collected anonymously, with participants providing informed consent at the beginning of the online survey before proceeding. Participant identifiers were sequentially assigned by LimeSurvey (e.g., first participant assigned ID 1, second ID 2, etc.), ensuring no personally identifiable information was retained. The survey introduction clearly informed participants that their participation was voluntary, anonymous, and confidential, and that their responses would be used for research purposes only. Participants were also informed they could stop the study at any time. The datasets contain no sensitive personal information, with demographic data limited to age, gender, profession, country details, and language information, none of which can be used to identify individual participants.

### 2.8. Existing Use of Data

Small parts of the dataset have been published or submitted for publication, namely associations with i) three colour terms in French (Epicoco et al., 2021, 2024), ii) 12 colour terms in English (Jonauskaite et al., in revision), and iii) three colour terms in six languages (Epicoco, et al., in prep.). We also proposed a coding system for free associations in Epicoco et al. (2021, 2024), inspired by the works of Rosch (1973) and Griber et al. (2018).

## 3. Dataset Description and Access

### 3.1. General Overview of The Dataset

**Figure 1:**
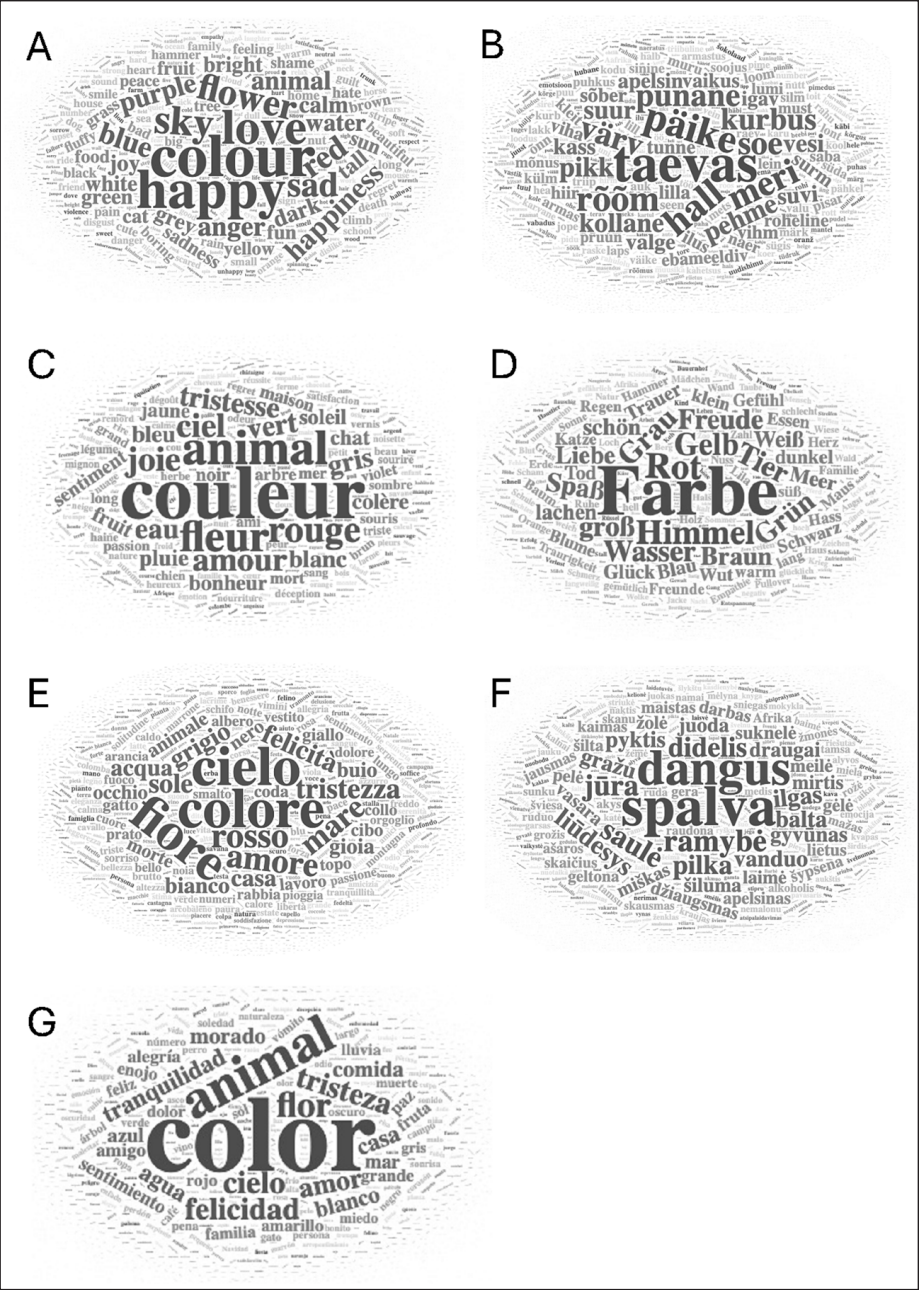
Word clouds in English **(A)**, Estonian **(B)**, French **(C)**, German **(D)**, Italian **(E)**, Lithuanian **(F)**, and Spanish **(G)**. Larger words indicate more frequent responses in the overall dataset of each language.

Our participants produced 223,786 responses in total. On average, there are 31,969 responses per language and 516 responses per stimulus word (see Table 4). We also determined the number of discrete responses per language and per stimulus word. By discrete responses, we refer to an ‘idea’, thus, ignoring how often an idea was produced. For instance, if the response COLOUR was produced 100 times in a language-specific dataset, COLOUR constituted a single discrete response (see Table 4). We report discrete responses for each language-specific dataset. To have a general overview of the obtained responses, in Figure 1, we display all the discrete responses in the form of word clouds, separately for each language. Larger font sizes indicate more frequent discrete responses.

**Table 4:** The number of participants, total responses and total discrete responses per language, across all stimulus words (total) and on average per stimulus word.


SURVEY LANGUAGE	PARTICIPANTS	RESPONSES		DISCRETE RESPONSES	
	
		TOTAL	PER STIMULUS WORD	TOTAL	PER STIMULUS WORD

English	241	33,415	538.95	10,757	173.50

Estonian	123	17,946	289.45	8,242	132.94

French	316	51,441	829.69	12,501	201.63

German	357	61,528	992.39	15,417	248.66

Italian	56	9,190	148.23	4,773	76.98

Lithuanian	179	24,008	387.23	9,754	157.32

Spanish	167	26,258	423.52	8,924	143.94

All languages	1,439	223,786	3,609.45	70,368	1,134.97


### 3.2. Repository Location

The research material and the data are accessible on OSF: https://osf.io/xzcbg/. The project is identified with the DOI: https://doi.org/10.17605/OSF.IO/XZCBG.

### 3.3. Object and File Names

The research material and the datasets are classified in the following folders:

– **Datasets**
  – **Cleaned data in seven languages**: Contains cleaned and normalised data in seven languages (one file per language, plus one file with all languages, where stimulus words are direct translations). Each participant’s data span several rows. Each cleaned participant response appears on a separate row, and demographic data are repeated across the rows. The column ‘Association number’ indicates whether this was their first, second, third, etc. response. Compared to the raw and uncleaned datasets, we recoded language and country codes using the international standardisation codes. *This dataset is recommended for analyses*.
  – **Raw data – all languages together**: Contains a single file with all the raw data. The file was downloaded directly from LimeSurvey and was not modified. Each participant’s data occupy a single row. The file includes data from all participants, including those excluded from the final dataset during the cleaning procedure (see Sampling, Sample, and Data Collection). This dataset can be matched with the other datasets using participant ID (Response ID -> PS_ID).
  – **Uncleaned data in seven languages**: Contains uncleaned data in seven languages (one file per language except for German, which has two files – see the explanation under Surveys). Each participant’s data span multiple rows, and demographic data are repeated. Each word a participant produced appears in a new row, in the order they had been typed. Words were extracted automatically, meaning that responses with more than one word appear on separate rows (e.g., *light blue* would be separated into two rows with *light* and *blue* as responses). Such errors were corrected during the cleaning process. This dataset can be matched with the other datasets using participant ID (Response ID -> PS_ID).
– **Stimuli and Codebook**
  – **Codebook_cleaned_dataset_variables.csv**: Contains variable names and their descriptions for the cleaned datasets.
  – **Codebook_datasets.csv**: Contains dataset organisation information, to help readers navigate the files (repeats some information from above).
  – **Stimulus_word_translations.csv**: Stimulus words in all seven languages (identical to those in Tables 2 and 3).
  – **Convert raw to uncleaned.R:** a custom R code, converting the raw data to the uncleaned data.
  – **Surveys**: Contains LimeSurvey PDFs in seven languages. There are two files for German: German_1 contains *Geld* (‘money’), and German_2 contains *Gelb* (‘yellow’) as stimulus words.

### 3.4. Data Type

The folder ‘Surveys’ includes research materials – LimeSurvey surveys in all seven languages. The folder ‘Raw data – all languages together’ includes primary raw and unprocessed data. The folder ‘Uncleaned data in seven languages’ includes primary data, after the first processing step. The folder ‘Cleaned data in seven languages’ includes primary processed data, after the data cleaning and normalisation. Generally, we recommend researchers in psychology to use data from the ‘Cleaned data in seven languages’ folder, unless their research question requires the raw data (e.g., studies examining the typing choices of respondents, typos, exact word forms, etc.).

### 3.5. Format Names and Versions

The folder ‘Surveys’ includes research materials in PDF format, that can be opened with standard document readers (e.g., Adobe Acrobat Reader, Preview, etc.). The folders under ‘Datasets’ include unprocessed and processed datasets in the XLSX and CSV formats. The XLSX files can be opened with Microsoft Excel or free alternatives, such as LibreOffice Calc, OpenOffice Calc, or Google Sheets, while the CSV files can additionally be opened with any text editor. Both, XLSX and CSV files can also be directly imported into R, Python, Matlab, SPSS, Stata, etc. The stimulus words in all seven languages (see Stimuli) and the relevant codebooks (see FAIR Data and Codebook) appear in ‘Stimuli and Codebook’ folder, in CSV format.

### 3.6. Language

Variable names that are common across the languages (e.g., participant ID, country of residence, country of origin, etc.) are displayed in British English. The stimulus words are displayed in the language of the survey – English, Estonian, French, German, Italian, Lithuanian, or Spanish. Participant responses are also displayed in the language of the survey.

### 3.7. Licence

The data are published under the CC-BY 4.0 license.

### 3.8. Limits to Sharing

There is no identifying information in the dataset. All participant IDs were generated by LimeSurvey in the order that participants took the survey. Therefore, there are no barriers to the full sharing of the dataset, which is fully accessible with no restrictions. It is not under embargo.

### 3.9. Publication Date

The data were published in the repository on 2^nd^ June 2025.

### 3.10. FAIR Data and Codebook

Our dataset adheres to the FAIR (Findability, Accessibility, Interoperability, and Reuse) principles in the following way (Wilkinson et al., 2016). For Findability, we have assigned a persistent DOI through the OSF repository, mentioned in the Repository location section. Accessibility is ensured through the open access, described in the Limits to Sharing section, and the choice of the data repository (OSF), which guarantees data preservation and long-term access. Interoperability is supported through the XLSX format, which is machine-readable and compatible with diverse software. Reusability is facilitated through the data cleaning and the file documentation, outlined in the Data Cleaning and Normalisation and Object and File Names sections respectively.

The dataset codebook, available as ‘Codebook_cleaned_datasets.xlsx’ in the repository, supplements the file structure information provided in the Object and File Names section. It contains complete descriptions of all variables including participant demographics (as referenced in the Sampling section), stimulus information (detailed in the Stimuli section), and response data (described in the General overview of the dataset section). The codebook also provides specific notes on language-specific features that complement the information provided in the Language section. The codebook ‘Codebook_datasets.xlsx’ facilitates navigation of the different datasets.

## 4. Reuse Potential

This dataset has substantial reuse potential across multiple disciplines and research contexts. It is a cross-linguistic dataset of free associations from seven languages. Its detailed documentation and standardised methodology make it an accessible and valuable resource for researchers investigating the intersection of language, thought, and culture. The primary strength lies in its comprehensive nature: it contains responses from 1,439 participants across 14 countries to 62 stimulus words, yielding a rich cross-linguistic repository of semantic associations. It can help investigate diverse research questions in various disciplines. Thus, our suggestions below are indicative, but not exhaustive. The data have been cleaned and normalised, making them ready for analysis.

### 4.1. Cognitive Psychology and Psycholinguistics

This dataset is useful in the study of semantic networks and meaning construction within and across languages and cultures. It is particularly useful for those interested in colour and emotion terms (e.g., see colour semantics in Schloss, 2024), and the interplay between the two (e.g., see Jonauskaite & Mohr, 2025; Ram et al., 2020). The dataset includes associations for basic and non-basic colour terms enabling a better understanding of how colour knowledge is organised (e.g., Epicoco et al., 2024; Mervis & Roth, 1981; Mylonas & Griffin, 2020). One can also compare associations for the same stimulus word across dialects or different language communities (e.g., Epicoco et al., 2021, 2024). Perhaps, such studies might help understanding historical and post-colonial influences (Levisen & Sippola, 2019; Wassmann, 2017) or contribute to anthropological research examining cultural values and meaning systems (Handwerker, 2002). This dataset supports the decolonisation of research practices in psychology by providing a valuable cross-linguistic resource, enabling researchers to move beyond an Anglocentric approach. Then, researchers can study and compare semantic universals and language-specific conceptualisations, like those relevant to idiomatic and metaphoric expressions (e.g., Oguz, 2025). Yet, analysis can go beyond colour and emotion terms, exploring the semantic structure of other natural and human-made categories that are part of the stimulus word set.

The cleaned and the raw datasets allow the analysis of response patterns. Using the cleaned data, researchers can tap into divergent thinking and creativity, for which spontaneous cognition and associative flexibility are central (Beaty & Kenett, 2023; Rossmann & Fink, 2010). Free association data can be analysed for fluency, originality, semantic distance and other features (e.g., Abu-Akel et al., 2020; Beaty et al., 2022; Merseal et al., 2025). The raw dataset shows variations in i) how words were typed (lowercase, capital letters, errors), ii) how language was used (e.g. dialect, foreign terms), and iii) other intra- and inter-individual variations, all of which yield information on behavioural thought patterns. Researchers could further reduce the complexity of the datasets by compiling participants’ responses into smaller entities. Epicoco et al. (2024) proposed a coding scheme of nine semantic themes, inspired by the earlier studies (Paramei et al., 2018; Rosch, 1973). Showing the potential of this approach, we previously applied this coding scheme to analyse i) the French colour terms *violet, lilas*, and *pourpre* in three countries (Epicoco et al., 2021, 2024) and ii) the reasons for colour preferences (Epicoco, et al., in revision).

### 4.2. Clinical and Developmental Psychology

This dataset could also serve as normative data for clinical research investigating semantic processing in various clinical populations such as schizophrenia, autism spectrum disorders, or depression (e.g., Pintos et al., 2022). For developmental research, data from younger age groups could be added (see Cox & Haebig, 2022) to examine how semantic knowledge develops from childhood through adulthood and old age (e.g., Citraro et al., 2023; Fourtassi et al., 2020; Unger & Fisher, 2021), or how associative patterns differ between native and non-native speakers to identify language learning trajectories (e.g., Kan et al., 2024). Shifts in emotional and non-emotional meaning-making could be detected, such as developmental trends in the representation of key affective concepts like *love* or *anger* (Nook et al., 2018). On the other end of the age continuum, researchers could investigate cognitive ageing directly within the current dataset, as we recruited participants from 16 to 80 years old (e.g., Jonauskaite et al., 2024).

### 4.3. Translation Studies

This dataset enables cross-linguistic analysis of semantic associations across Indo-European languages (English, French, German, Italian, Lithuanian, Spanish) and a Finno-Ugric language (Estonian). It can provide insights into translation theory and practice (Baker, 2018). Professional translators could benefit from this resource such as when having to i) translate connotative meanings of notoriously difficult to translate words (e.g., Uusküla, 2020; Wassmann, 2017) or ii) dealing with cognitive processes underlying translation difficulties in idiomatic language (e.g., Kalda & Uusküla, 2019; Oguz & Uusküla, 2023).

### 4.4. Education

Free association patterns could support the development of teaching materials that reflect how native speakers conceptually organise vocabulary (e.g., Curry & Mark, 2024; Looi et al., 2025). Knowing the most common associations that native speakers make with particular words, language instructors could design vocabulary learning activities that are supported by cognitive organisation rather than arbitrary word lists (e.g., Kismetova & Serikova, 2024). The cross-linguistic nature of the dataset is useful for comparative language education to highlight similarities and differences in how concepts are linked within and across languages (e.g., Ivanova, 2024).

### 4.5. Marketing

Free associations could help understand the emotional, symbolic, and otherwise abstract meaning of products, brands, and services in consumer contexts (Rahman & Areni, 2016; Vriens et al., 2019). Analysing the affective and sensory associations of personal experiences and representations might help making advertisements more memorable and personally relevant (see also Sester et al., 2013). The dataset incorporates different demographic variables and encompasses participants from a wide age range. Hence, the current data might help tailor messages to selected audiences and causes (Olsen & Pracejus, 2020; Yang & Li, 2016).

### 4.6. Limitations and Considerations for Reuse

Potential users should consider several limitations when reusing this data. While the sample size is substantial (*N* = 1,439), the distribution across languages is uneven, with larger samples for i) French and German, and ii) women than men. The sample sizes across the different countries are also uneven. Then, French and German samples had more young university students than samples in the other languages, potentially limiting age and socio-economic diversity (of which we have no information). Finally, data collection occurred between 2019 and 2023, which could introduce temporal effects, as semantic associations are constantly evolving (e.g., Unger & Fisher, 2019).

### 4.7. Future Potential

This dataset can serve as a foundation for future projects. It could be expanded to include additional languages, used in longitudinal studies that track changes in semantic associations over time, combined with neuroimaging studies to investigate the neural correlates of cross-linguistic semantic processing, and integrated with other cross-cultural datasets to examine broader patterns of cognition. The dataset could also be used as training and testing data for computational linguistics or to compare with the free associations generated by large language models (see Abramski et al., 2025). It could support the development and evaluation of semantic models across multiple languages, improvement of machine translation systems by incorporating culturally specific semantic associations, enhancement of natural language understanding in multilingual contexts, and testing of computational models of semantic networks. The cleaned, normalised format makes this dataset immediately usable for computational applications without extensive pre-processing. By providing well-constructed, cross-linguistic datasets, it encourages decolonisation in the research practice.

## Footnotes

Our subjective measure of language fluency gives some indication of how well participants understood the language of the survey. Yet, self-report measures are prone to biases (e.g., Hernández-Rivera et al., 2024). Thus, this score should be interpreted with caution.
